# Supplementary material for: Deep, Staged Transcriptomic Resources for the Novel Coleopteran Models Atrachya menetriesi and Callosobruchus maculatus
Source: PLoS One. 2016 Dec 1;11(12):e0167431. doi: 10.1371/journal.pone.0167431 (PMC5132259; doi:10.1371/journal.pone.0167431)
Supplement: S6 File — (GZ) [file pone.0167431.s006.gz › AdditionalFile6AtrachyaDiffExp.zip!/AdditionalFile8AtrachyaDiffExp/isoforms.counts.matrix.48_72_vs_72_100.edgeR.DE_results.MA_n_Volcano.pdf]

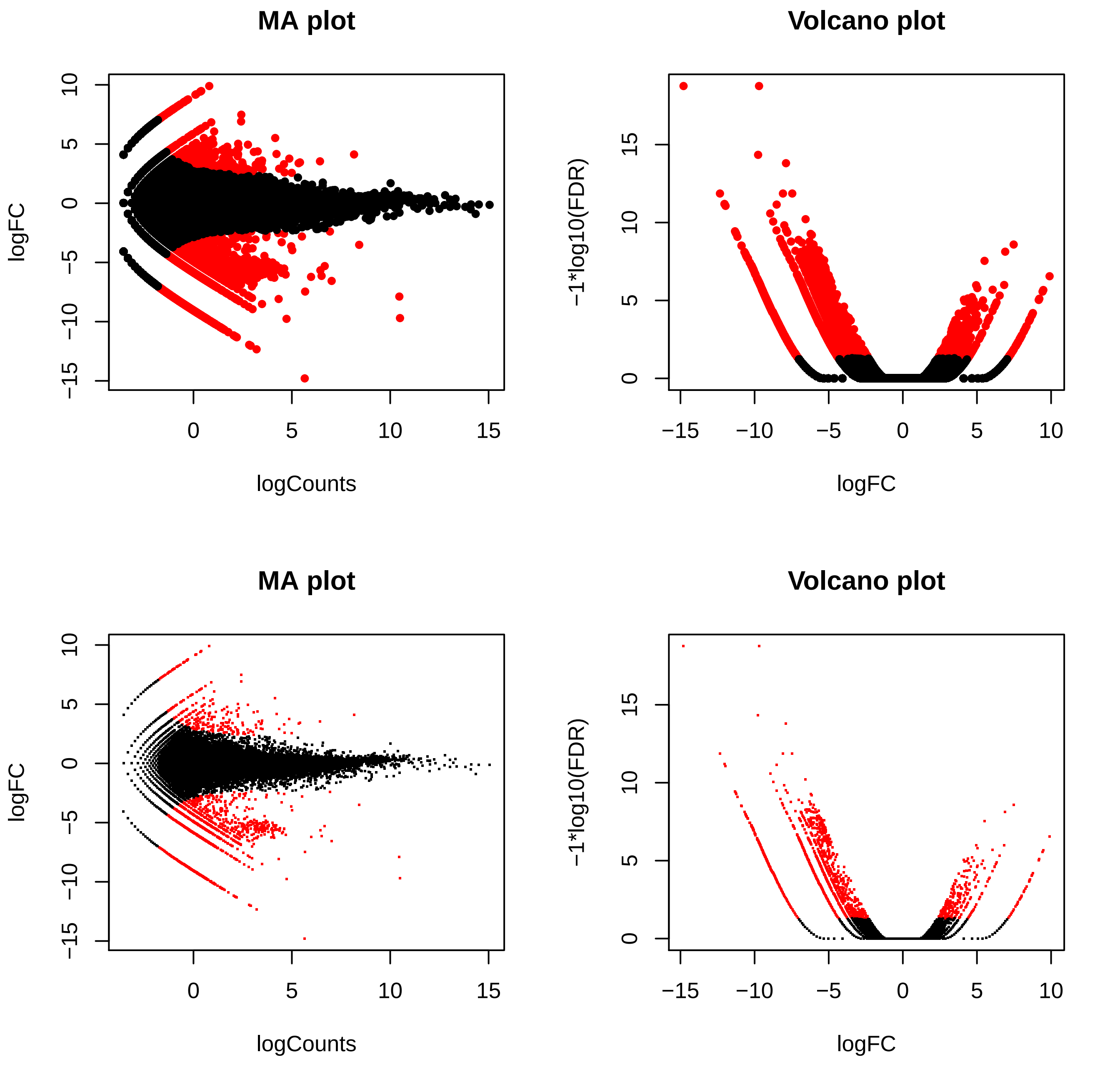
MA plot
logFC
logCounts
10
5
0
−5
−10
−15
0
5
10
15
Volcano plot
−1*log10(FDR)
logFC
15
10
5
0
−15
−10
−5
0
5
10
MA plot
logFC
logCounts
Volcano plot
−1*log10(FDR)
logFC
