# Supplementary material for: Deep, Staged Transcriptomic Resources for the Novel Coleopteran Models Atrachya menetriesi and Callosobruchus maculatus
Source: PLoS One. 2016 Dec 1;11(12):e0167431. doi: 10.1371/journal.pone.0167431 (PMC5132259; doi:10.1371/journal.pone.0167431)
Supplement: S6 File — (GZ) [file pone.0167431.s006.gz › AdditionalFile6AtrachyaDiffExp.zip!/AdditionalFile8AtrachyaDiffExp/isoformsclustered.matrix.log2.centered.genes_vs_samples_heatmap.pdf]

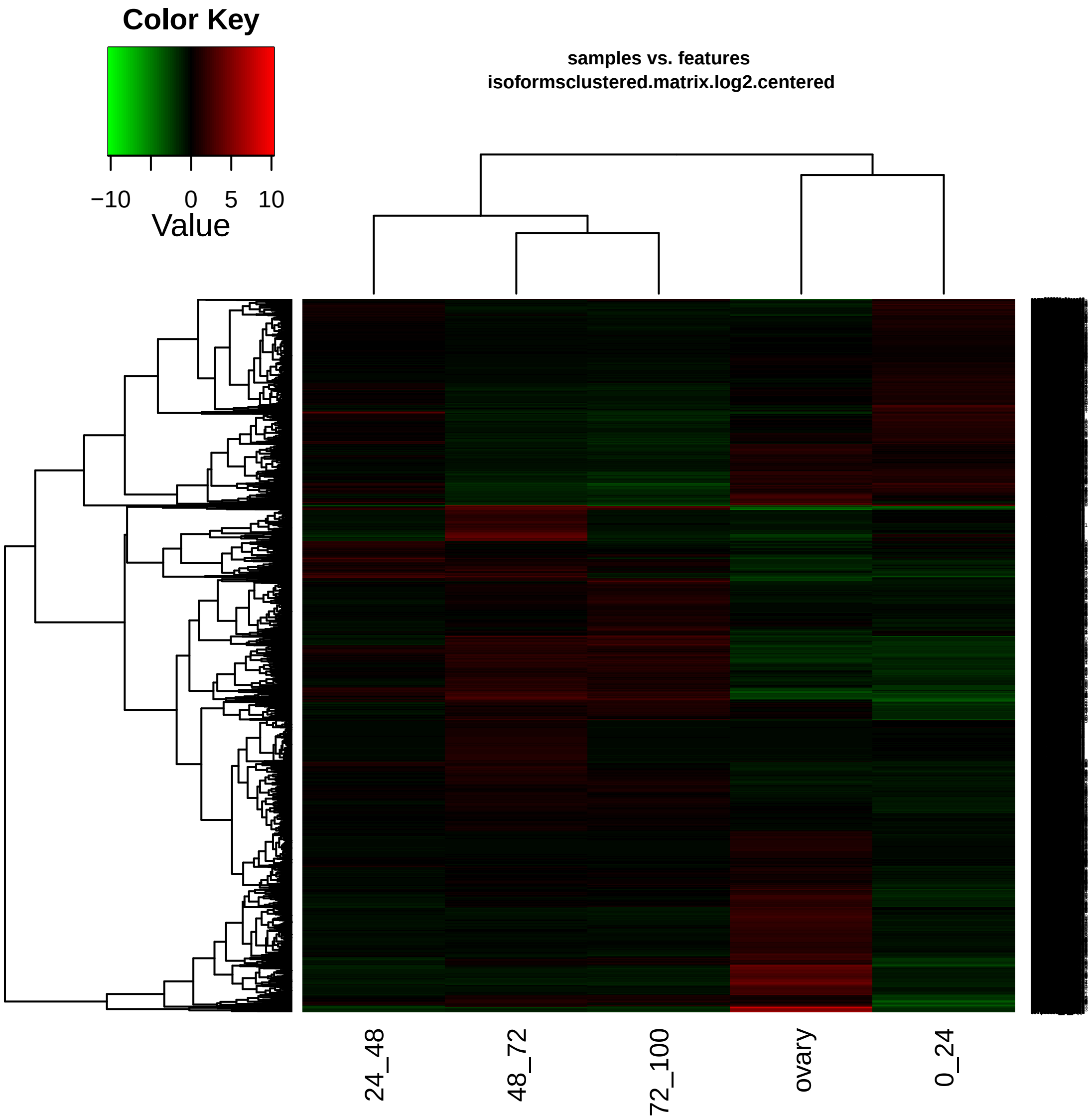

Color Key
−10
0
5
10
Value
samples vs. features
isoformsclustered.matrix.log2.centered
24_48
48_72
72_100
ovary
0_24
